# Decreased Netrin-1 and Correlated Th17/Tregs Balance Disorder in Aβ_1–42_ Induced Alzheimer’s Disease Model Rats

**DOI:** 10.3389/fnagi.2019.00124

**Published:** 2019-05-28

**Authors:** Lina Sun, Ting Ju, Tianhang Wang, Liang Zhang, Feifan Ding, Yan Zhang, Ran An, Yilei Sun, You Li, Yidan Lu, Xin Zhang, Lijun Chi

**Affiliations:** Department of Neurology, The First Affiliated Hospital of Harbin Medical University, Harbin, China

**Keywords:** Alzheimer’s disease, inflammation, netrin-1, Th17, Tregs

## Abstract

There is increasing evidence indicating that inflammation represents a key pathological component of Alzheimer’s disease (AD). A possible factor that may contribute to this process is netrin-1, a neuronal guidance molecule. This molecule has been shown to exert an unexpected immunomodulatory function. However, the potential changes and correlations of netrin-1 with T helper 17/regulatory T cells (Th17/Tregs) as related to inflammation in AD has yet to be examined. In this study, netrin-1 and Th17/Tregs balance were investigated, and the relationship among netrin-1, Th17/Tregs and cognitive function were analyzed in a rat model of AD. In this model, a bilateral intracerebroventricular administration of Amyloid β_1-42_ (Aβ_1–42_) was used to produce spatial learning and memory deficits, as well as increased neuronal apoptosis, which were detected 7 days after injection for AD7d group and 14 days for AD14d group. Netrin-1 concentrations were significantly down regulated in both serum and cerebrospinal fluid (CSF) of these AD rats, effects which were strongly correlated with cognitive deficits. Increased levels of interleukin (IL)-17 and deceased IL-10 were observed in both the circulation and CSF and were also correlated with the percent of time spent in the target quadrant of AD in these rats. These changes resulted in netrin-1 concentrations being negatively correlated with IL-17 but positively correlated with IL-10 concentrations in the serum and CSF. We also found that the Th17/Tregs balance was disrupted in these AD rats. Collectively, these findings reveal that the reduction in netrin-1 and the correlated disruption of Th17/Tregs balance in AD rats may diminish the immunosuppressive effect of netrin-1 on Th17/Tregs in AD pathogenesis.

## Highlights

- Netrin-1 was decreased in the serum and cerebrospinal fluid of AD rats.
- Th17/Tregs balance was disrupted and correlated with netrin-1 in AD rats.
- Decreased netrin-1 and correlated Th17/Tregs balance disorder were strongly correlated with cognitive dysfunction in AD rats.

## Introduction

Alzheimer’s disease (AD) is the foremost cause of dementia worldwide (Prince et al., 2015). While the pathogenesis of AD is multifactorial, inflammation has attracted considerable attention of late as being a key pathological component in the development of this disease (Sun et al., 2013; Van Eldik et al., 2016). For example, the accumulation of neurotoxic amyloid beta (Aβ) oligomeric peptides leads to proinflammatory cytokines in the preclinical phases of AD, effects which could possibly be counterbalanced by anti-inflammatory mechanisms (Lee et al., 2016; Cuello, 2017). Activation of glial cells, such as microglia, were commonly considered to initiate this inflammation process. However, T lymphocytes have also been shown to participate in AD-related neuroinflammation and outcomes (Town et al., 2005).

Generally, few activated T lymphocytes patrol the central nervous system (CNS), however, under inflammatory conditions peripheral lymphocytes infiltrate across the blood-brain barrier into the parenchyma. Notably, T helper 17 (Th17) cells and regulatory T cells (Tregs) are two subsets of CD4+ T lymphocytes distinct from traditional Th1 and Th2 cells. Th17 cells are highly proinflammatory, secreting interleukins (IL)-17, IL-21, IL-22, and IL-23 (Maddur et al., 2012); and it has been demonstred *in vitro* that the Th17 cytokines, IL-21 and IL-22, and the specific transcriptional factor RORγ were significantly increased in Aβ stimulated T lymphocytes of AD patients (Saresella et al., 2011). Moreover, in the Aβ_1–42_ induced rat model of AD, Th17 cells infiltrated into brain parenchyma, generating IL-17 and IL-22, and directly acted upon neurons through the Fas/FasL apoptotic pathway (Zhang et al., 2013). Such findings indicate a participation of Th17 cells in AD pathology.

Tregs, which mainly produce transforming growth factor-β (TGF-β) and IL-10, play a pivotal role in maintaining immunological homeostasis and regulating autoimmunity (Sakaguchi et al., 2010). Findings from recent studies using rodent models have indicated that Tregs may exert a protective effect in AD pathogenesis. The Tregs cytokines, TGF-β and IL-35, along with the specific transcriptional factor Foxp3+, were all found to be decreased in the cortex and hippocampus of the Aβ_1–42_ induced rat model of AD (Zhang et al., 2015). In the transgenic mouse model of AD, systemic transplantation or amplification of Tregs improved cognitive functions (Yang et al., 2013; Baek et al., 2016). Moreover, a temporary depletion of Tregs furthered initial learning impairments in APPPS1 mice (Dansokho et al., 2016) and aggravated cognitive deficits of 3xTg-AD mice (Baek et al., 2016). Accordingly, there appear to be significant roles for Th17/Tregs in AD pathogenesis that warrant further investigation.

Netrin-1, a classical bi-functional axonal guidance molecule, has been shown to be involved in a number of pathophysiological processses. In particular, its role in inflammation has made it an attractive and important molecule of emerging research interest (Ly et al., 2005; Rajasekharan and Kennedy, 2009). In the periphery, netrin-1 alleviates inflammation induced by hypoxia and acute lung injury through its capacity to inhibit leukocyte infiltration and cytokine production (Rosenberger et al., 2009; Mutz et al., 2010; Valbona et al., 2010). Netrin-1 also suppressed Th1/Th2/Th17 cytokine production from CD4+T cells as demonstrated *in vitro*, and protected the kidney against ischemia-reperfusion injury through the UNC5B receptor as shown *in vivo* (Tadagavadi et al., 2010). Interestingly, overexpression of netrin-1, which is present in the mouse model of atherosclerosis, was associated with a robust up regulation of Tregs, and reduced monocytes/macrophages accumulation and subsequent plaque formation (Khan et al., 2011).

With regard to neurological conditions, netrin-1 possesses neuroprotective functions involving anti-inflammatory effects as demonstrated in multiple sclerosis (MS) (Mulero et al., 2017), experimental autoimmune encephalitis (EAE) (Podjaski et al., 2015) and subarachnoid hemorrhage (SAH) (Xie et al., 2017b). Netrin-1 was significantly increased within the serum of MS patients as compared to controls and blood vessels of perivascular lesions within MS patients as well as in EAE mice, which showed increased expressions of netrin-1 within the brain. Within EAE mice, an early administration of recombinant netrin-1 delayed disease onset and restored disease scores in EAE mice, effects which may likely be related to redutions in inflammatory lesions and proportions of Th17 in the CNS (Podjaski et al., 2015). An exogenous administration of netrin-1 also protected against brain injury by activation of anti-apoptotic mechanisms. In addition, netrin-1 preserved blood–brain barrier integrity in SAH (Xie et al., 2017a,c), diminished brain injury secondary to stroke (Wu et al., 2008; Liao et al., 2013; Yu et al., 2017) and intracerebral hemorrhage (Wang et al., 2017). In animal models of AD, netrin-1 has been reported to reduce Aβ_1–40_-induced Aβ_1–42_ increases, as demonstrated *in vitro*, and decrease both Aβ_1–40_ and Aβ_1–42_ in PDAPP^Swe/Ind^ transgenic mice, effects which were accompanied with improved working memory (Spilman et al., 2016). However, whether these effects of netrin-1 may be related to that of Th17/Tregs inflammation in AD has yet to be examined.

Given this background information regarding netrin-1, together with the critical role of inflammation in AD, on the basis of behavioral and immunohistochemical changes detection, in the present report we focussed upon three primary goals. The first goal was to determine netrin-1 levels in both the serum and cerebrospinal fluid (CSF) within a rat model of AD. Second, to determine the balance of Th17/Tregs in this model. Finally, to examine the relationships between netrin-1 and Th17/Tregs and their possible roles in AD pathology.

## Materials and Methods

### Animals and Ethics Statement

Adult male Sprague Dawley rats (weight 300–350 g) were obtained from the Center of Experimental Animals at Harbin Medical University, China. The rats were housed in a room maintained on a 12-h-light/dark cycle with constant temperature and humidity and permitted food and water *ad libitum*. All experimental protocols in this study were approved by the ethics committee of Harbin Medical University.

### Induction of the Aβ_1–42_ Induced Rat Model of AD

The rats were randomly divided into three groups: control, AD7d, AD14d (*n* = 9, respectively). Aβ_1–42_ (Sigma-Aldrich, United States) was dissolved in sterilized saline (1 mg/mL) and then incubated at 37°C for 7 days (Zhang et al., 2013). Animals were deeply anesthetized with a 10% chloral hydrate solution of 3 ml/kg and mounted in a stereotactic frame. Five μL of Aβ_1–42_ (1 μg/μL) were slowly injected into each lateral ventricle with a microsyringe using the following stereotaxic coordinates of the rat brain atlas: 0.8 mm posterior to bregma, 1.5 mm lateral to the sagital suture, and at a depth of 3.5 mm from the surface of the brain (Paxinos and Watson, 2005; Frozza et al., 2013). Equal volumes of saline were administrated identically into Control rats. Animals were subjected to behavioral tasks at either 7 days (Control, AD7d group) or 14 days (AD14d group) after Aβ_1–42_ injection.

### Morris Water Maze Test

**FIGURE 1:**
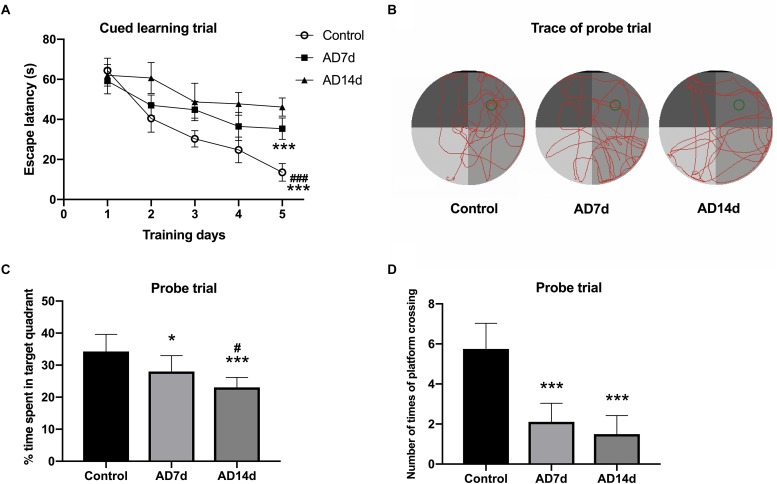
Administration of Aβ_1–42_ induces cognitive impairment. Following Aβ_1–42_ injection, the three groups of rats (7 days for Control and AD7d group, 14 days for AD14d group) were evaluated for spatial learning and memory in the MWM test. **(A)** Mean daily latencies to escape from the start point onto the hidden platform in the cued learning trail. **(B)** Representative path traces during the probe test. **(C)** Percent of time spent in the target quadrant relative to total time in the pool during the probe trial. **(D)** Number of crossings of the target platform during the probe trial. *n* = 9 per group, mean ± standard deviation, ^∗^*P* < 0.05, ^∗∗∗^*P* < 0.001 vs. Control group, group. ^#^*P* < 0.05, ^###^*P* < 0.001 vs. AD7d.

The Morris water maze (MWM) test was performed to assess spatial learning and memory as described in detail previously, with some alterations (Ai et al., 2013). The water maze consisted of a black circular tank (2.0 m diameter) filled with opaque water (23 ± 1°C) as achieved with the addition of black food pigment. An escape platform (20 cm diameter, 2 cm under the water surface) was located in the center of the first quadrant (indicated as a green circle in Figure 1B). Prior to training, rats with pupillary light reflex dysfunction were removed from the trials. In the cued learning trial, the rats received three trials per day for 5 consecutive days to locate the hidden platform. In these trials, the rats started from different quadrants, other than the target quadrant, with an alternation of 60 s swim and 30 s rest. If they failed to locate the platform within 120 s, the rats were gently guided to the platform and allowed to remain on the platform for at least 20 s. In the probe trial, rats were allowed to swim freely in the pool for 120 s with the platform removed on the sixth day. Escape latencies, percent of time spent in the target quadrant, and number of crossings of the platform were monitored by an online DigBehav-Morris water maze Video Analysis System (Mobile Datum Software Technology).

### TUNEL Staining and NeuN Immunolabeling

The brain was carefully removd and rapidly separated on ice with bone forceps immediately after sacrifice. Coronal hippocampal sections (40 μm thick) were collected with use of a freezing microtome and fixed in 4% paraformaldehyde in 0.01 mol/L phosphate-buffered saline (PBS, pH 7.3) for 30 min, followed by incubation with 0.3% Triton X-100 and 5% goat serum for 30 min. The slices were then incubated with rabbit anti-NeuN primary antibody (Proteintech, United States, 1:100) overnight at 4°C, washed three times with PBS and incubated with Cy3-conjugated affinipure goat anti-rabbit IgG (Proteintech, United States, 1:100) for 1 h at 37°C. To assess apoptosis, the TUNEL assay Kit (KeyGEN BioTECH, China) was used according to the manufacturer’s instructions. Briefly, after NeuN staining, hippocampal sections were incubated with TUNEL reaction mixture for 60 min at 37°C, with the exception of negative control sections. After washing with PBS, 50 μl streptavidin-fluorescein (diluted 1:9 with Labeling Buffer) was addedd to each slice for 30 min at 37°C in the dark, and then observed (six hippocampal sections per rat) under fluorescence microscopy. Totally, 18 visual fields in six sections of the CA1 hippocampal region per rat were manually counted for TUNEL and NeuN stained cells.

### Flow Cytometric Analysis

The frequency of Th17 and Tregs in CD4+ T cells in peripheral blood were determined with the use of flow cytometry as described previously with some modifications (Chi et al., 2007, 2010). Briefly, 200 μl of fresh peripheral blood obtained from caudal vein of each rat. The red blood cells were lysed and washed with PBS. Cells were initially stained extracellularly with use of phycoerythrin (PE) anti-human CD4 (eBioscience, United States) at 4°C for 20 min. Subsequently, cells were fixed and permeabilized, and stained with fluorescein isothiocyanate (FITC)-conjugated IL-17A (BioLegend, United States) for Th17 detection and PerCPCy5.5-conjugated IFN-γ (BioLegend, United States) for Th1 detection. For Tregs, the CD4-PE cells were stained with FITC-conjugated CD25 (BioLegend, United States). Flow cytometric analysis was performed with use of a fluorescence-activated cell sorter Calibur cytometer. Data were analyzed with CellQuest software (Becton Dickinson, United States).

### Enzyme-Linked Immunosorbent Assay

Enzyme-linked immunosorbent assay (ELISA) kits were used to quantify levels of netrin-1 (MEIMIAN, China), IL-17 (MEIMIAN, China) and IL-10 (RayBiotech, United States) in duplicate wells containing serum and CSF samples according to the manufacturers’ protocols. Blood was collected via a right ventricular puncture and about 1–2 ml serum per rat was obtained following centrifugation at 3000 rpm for 5 min. Approximately 100 μl of CSF was collected from each rat via foramen magnum puncture. Briefly, for detection of IL-10, samples were incubated for 2.5 h in a microtiter plate pre-coated with monoclonal antibody, and then for 60 min with biotinylated antibody at room temperature, followed by the addition of streptavidin solution for 45 min and one-step substrate reagent for 30 min as performed in the dark at 37°C. For netrin-1 and IL-17, each sample and its control were incubated in the 96-well microtiter plate for 0.5 h at 37°C, followed by HRP-conjugated reagent and chromogen solution at 37°C. All optical densities were measured at 450 nm. Each sample was detected in duplicated wells and the results were averaged.

### Statistical Analysis

Data are presented as the mean ± standard deviation and were analyzed using one-way analysis of variance followed by the LSD test for *post hoc* comparisons. The relationships among these molecules were evaluated with the use of linear regression and correlation analysis. A *P* < 0.05 was required for results to be considered statistically significant.

## Results

### Administration of Aβ_1–42_ Induces Cognitive Impairment

Following Aβ_1–42_ administration, the rats were evaluated (at 7 days for the Control and AD7d groups and at 14 days for the AD14d group) for spatial learning and memory in the MWM test. During the cued learning trail, escape latencies were markedly elevated for the rats in AD7d and AD14d groups as compared to the control group, as determined on the fifth day (Figure 1A). In the probe trial, the percent of time spent in the target quadrant was significantly decreased and the number of crossings of the previous target platform was also substantially decreased in AD rats (Figure 1B–D). The percent of time in the target quadrant and number of times of platform crossing were further decreased on day 6 in the AD14d versus AD7d group, suggesting a progression in the amount of neurodegeneration was present in the former. These data demonstrated that Aβ_1–42_ administration produced a clear decline in the learning and memory ability of these rats.

### Administration of Aβ_1–42_ Induces Neurotoxicity

**FIGURE 2:**
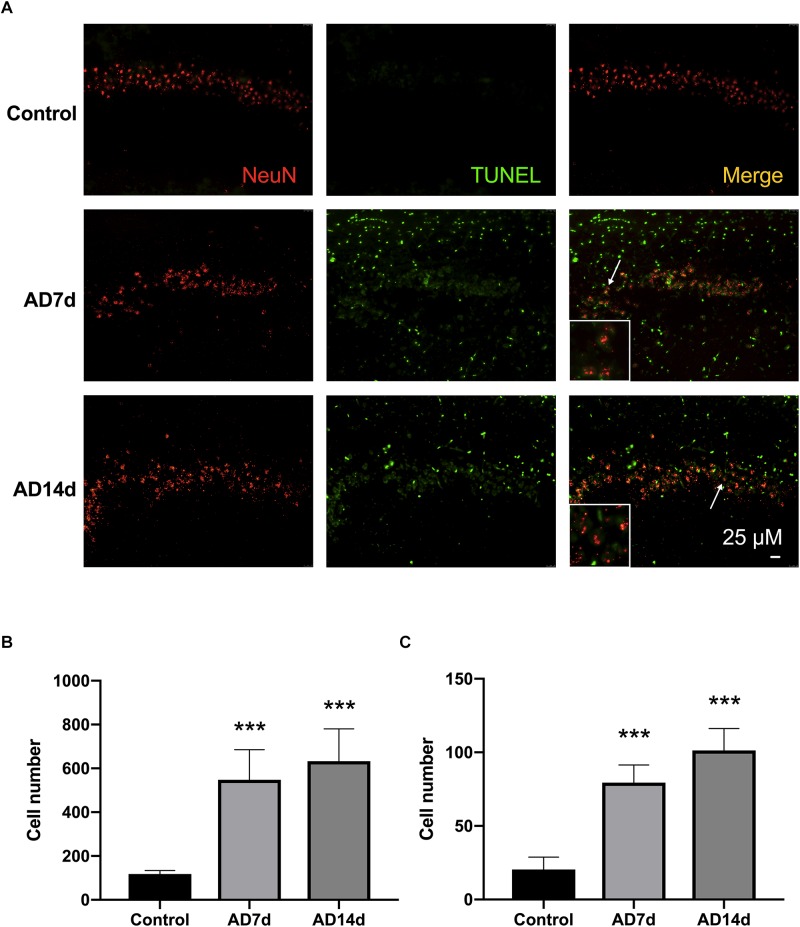
Administration of Aβ_1–42_ induces neurotoxicity. **(A)** Representative images of immunofluorescent histochemistry for NeuN and TUNEL in hippocampal CA1 regions of Control, AD7d, and AD14d groups. White arrows indicate TUNEL/NeuN double-stained cells. Scale bars: 25 μm. **(B)** Statistical column chart of of the number of TUNEL-stained cells. **(C)** Statistical column chart of the number of NeuN-TUNEL neurons. A total of 18 visual fields in six hippocampal sections were counted for the TUNEL-positive or TUNEL/NeuN double-stained cells in each rat. *n* = 7 per group, mean ± standard deviation, ^∗∗∗^*P* < 0.001 vs. Control group.

Results from immunofluorescent histochemistry of hippocampal sections, revealed that the number of TUNEL-stained cells increased within the CA1 region of AD rats as compared with that in Control rats. Moreover, the number of NeuN-TUNEL neurons were significantly increased in the AD7d and AD14d groups relative to the Control group, indicating that this Aβ_1–42_ administration induced neuronal apoptosis. No statistically significant differences were obtained between the AD7d and AD14d groups (Figure 2).

### Down-Regulation of Netrin-1 in the Serum and CSF of AD Rats

**FIGURE 3:**
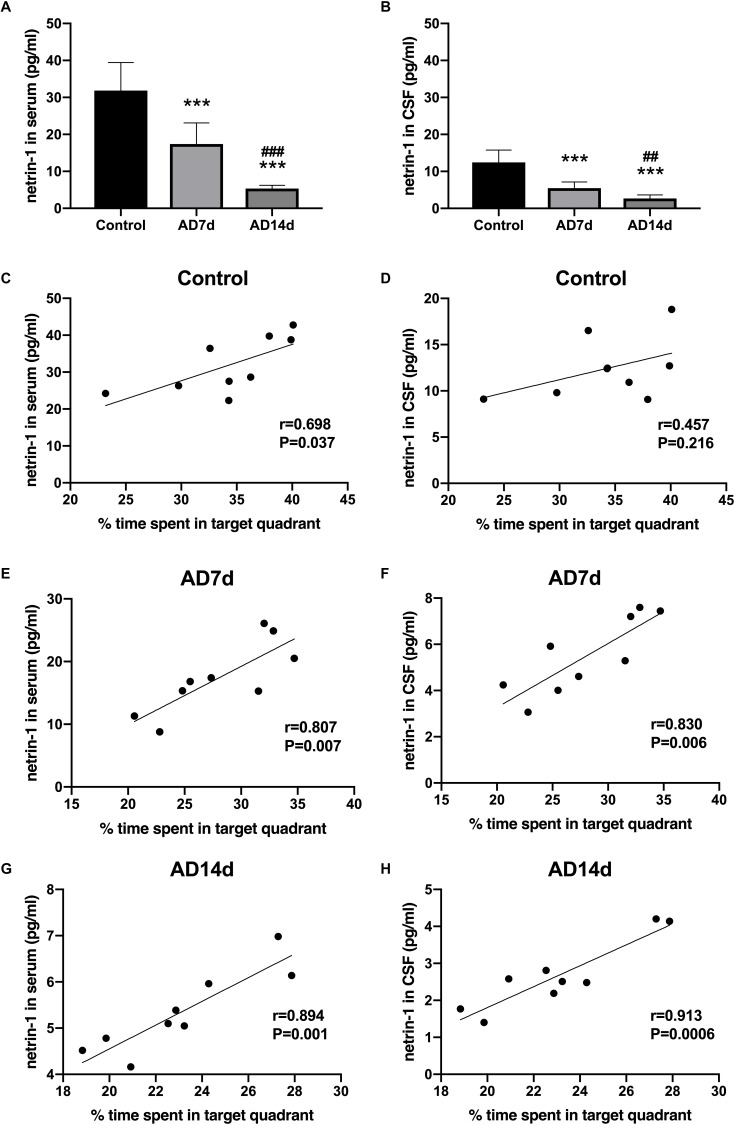
Down-regulation of netrin-1 in the serum and CSF of AD rats. Netrin-1 concentrations in the serum **(A)** and CSF **(B)** of the Control, AD7d and AD14d groups were determined with use of ELISA. *n* = 9 per group, mean ± standard deviation, ^∗∗∗^*P* < 0.001 vs. Control group, ##*P* < 0.01, ###*P* < 0.001 vs. AD7d group. Positive correlations were obtained between serum netrin-1 concentrations and percent of time in the target quadrant in the Control **(C)**, AD7d **(E)**, and AD14d **(G)** groups. Positive correlations were obtained between CSF netrin-1 concentrations and percent of time in the target quadrant in the Control **(D)**, AD7d **(F)**, and AD14d **(H)** groups.

To determine whether netrin-1 may be involved in AD progression, we assayed for levels of netrin-1 in both serum and CSF with the use of ELISA. As shown in Figure 3A,B, serum and CSF levels of netrin-1 were significantly down regulated in AD rats as compared with those in controls. More severe reductions in serum and CSF netrin-1 were observed in the AD14d group (5.342 ± 0.883 and 2.676 ± 0.952, respectively) than those in the AD7d group (17.382 ± 5.718 and 5.486 ± 1.652, respectively). Moreover, we found that serum and CSF concentrations of netrin-1 in AD rats were positively correlated with the percent of time within the target quadrant (Figure 3E–H), indicating a clear relationship between this molecule and spatial learning. Interestingly, netrin-1 levels in the serum, but not in the CSF, were also correlated with the percent of time in the target quadrant in the Control group (Figure 3C,D). These correlations were less robust than those obtained for AD rats. and this relationship was not present in determinations from the CSF.

### Increased IL-17 and Decreased IL-10 in the Serum and CSF of AD Rats

**FIGURE 4:**
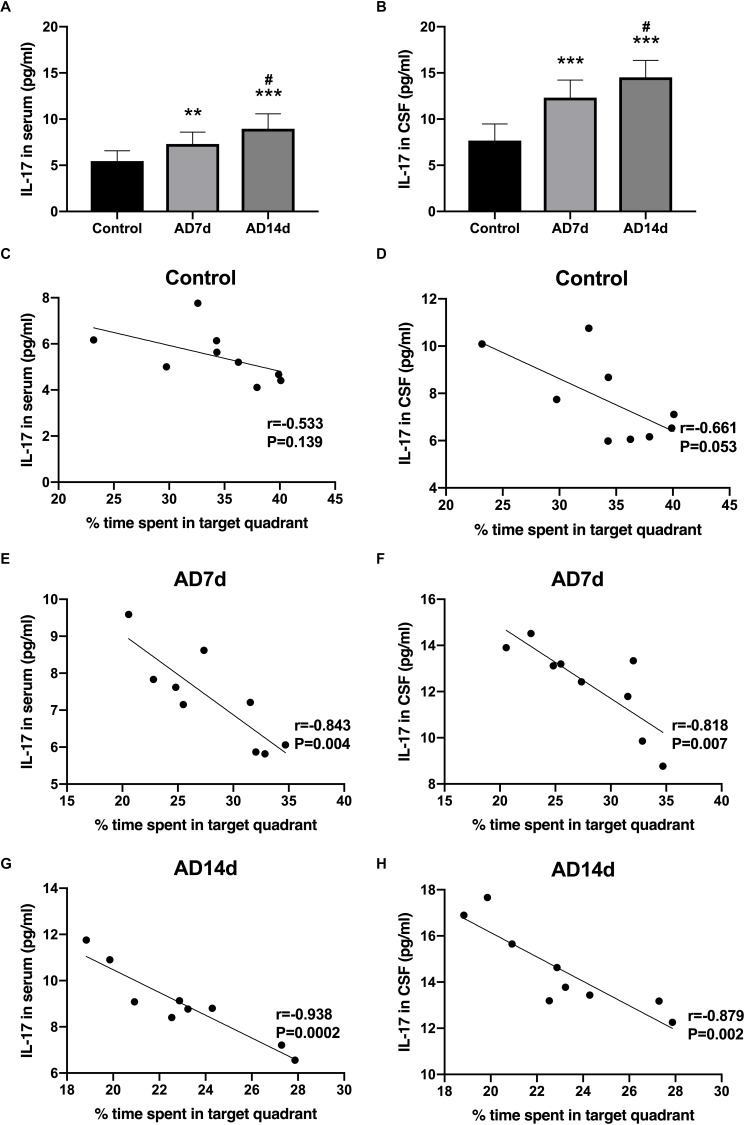
Increased IL-17 concentrations in the serum and CSF of AD rats. IL-17 concentrations in the serum **(A)** and CSF **(B)** of the Control, AD7d, and AD14d groups were determined with use of ELISA. *n* = 9 per group, mean ± standard deviation, ^∗∗^*P* < 0.01, ^∗∗∗^*P* < 0.001 vs. Control group, #*P* < 0.05 vs. AD7d group. Negative correlations were obtained between serum IL-17 concentrations and percent of time in the target quadrant in the Control **(C)**, AD7d **(E)**, and AD14d **(G)** groups. Negative correlations were obtained between CSF IL-17 concentrations and percent of time in the target quadrant in the Control **(D)**, AD7d **(F)**, and AD14d **(H)** groups.

**FIGURE 5:**
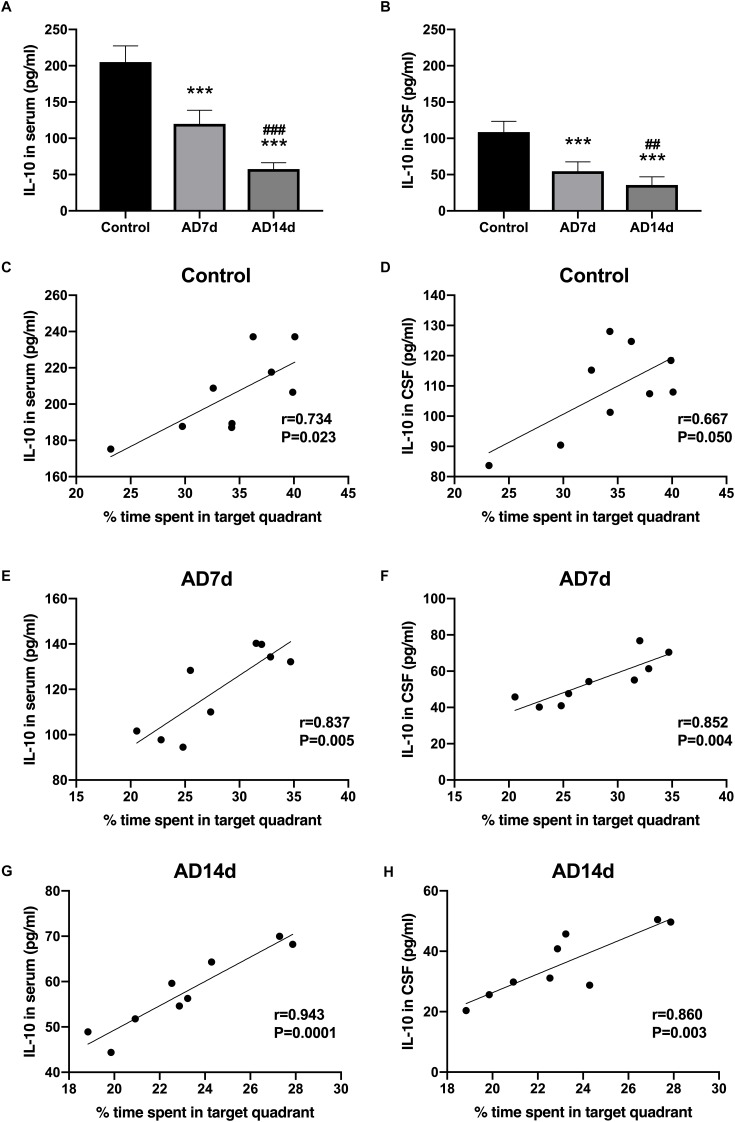
Decreased IL-10 concentrations in the serum and CSF of AD rats. IL-10 concentrations in the serum **(A)** and CSF **(B)** of the Control, AD7d and AD14d groups were determined with use of ELISA. *n* = 9 per group, mean ± standard deviation, ^∗∗∗^*P* < 0.001 vs. Control group, ##*P* < 0.01, ###*P* < 0.001 vs. AD7d group. Positive correlations were obtained between serum IL-10 concentrations and percent of time in the target quadrant in the Control **(C)**, AD7d **(E)**, and AD14d **(G)** groups. Positive correlations were obtained between CSF IL-10 concentrations and percent of time in the target quadrant in the Control **(D)**, AD7d **(F)**, and AD14d **(H)** groups.

IL-17 concentrations in both serum and CSF were elevated in AD rats (Figure 4A,B). In contrast, concentrations of one of the main Tregs cytokine, IL-10, were decreased in both the serum and CSF of AD rats (Figure 5A,B). Further elevations in IL-17 and further reductions in IL-10 concentrations were obtained within the AD14d as compared with the AD7d group. No statistically significant correlations were obtained between IL-17 concentrations in either the serum or CSF and the percent of time in the target quadrant within Control rats (Figure 4C,D). In contrast, serum IL-10 concentrations, but not CSF IL-10 concentrations, were positively correlated with the percent of time in the target quadrant in this group (Figure 5C,D). Within the AD rats, both serum and CSF IL-17 concentrations were negatively correlated with the percent of time in the target quadrant (Figure 4E–H), whereas serum and CSF IL-10 concentrations were positively correlated with the percent of time in the target quadrant (Figure 5E–H). Such correlations were more salient than that observed in the Control group.

### Th17/Tregs Balance Was Disrupted Within the Peripheral Blood of AD Rats

**FIGURE 6:**
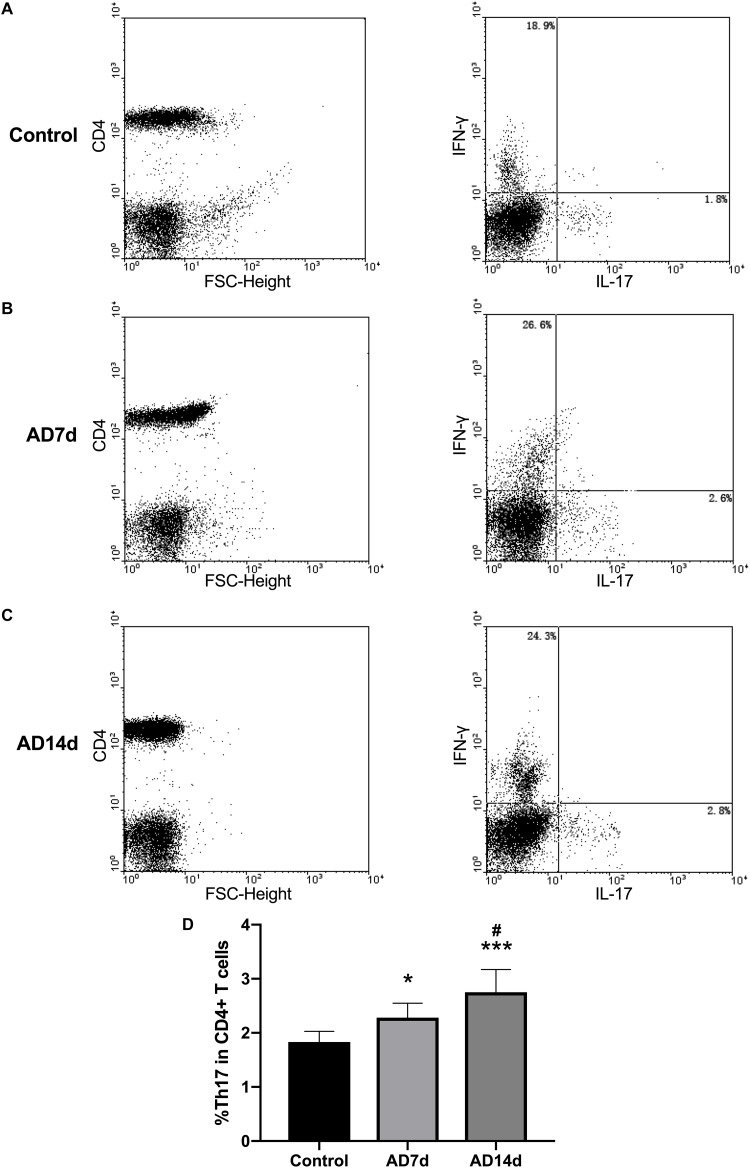
Elevated circulating Th17 populations in AD rats. The population of Th17, a subset percent of total CD4+ T cells, was estimated with use of flow cytometry. Dot plots shown are representative of rats in the Control **(A)**, AD7d **(B)**, and AD14d **(C)** groups. Th17 cells were confirmed as CD4^+^IL-17^+^IFN-γ^-^ cells. Numbers in each compartment indicate the proportions of Th17 and Th1 in CD4^+^ cells. **(D)** Percent of Th17 in total CD4+ T cells in the Control, AD7d and AD14d groups. *n* = 9 per group, mean ± standard deviation, ^∗^*P* < 0.05, ^∗∗∗^*P* < 0.001 vs. Control group, #*P* < 0.05 vs. AD7d group.

**FIGURE 7:**
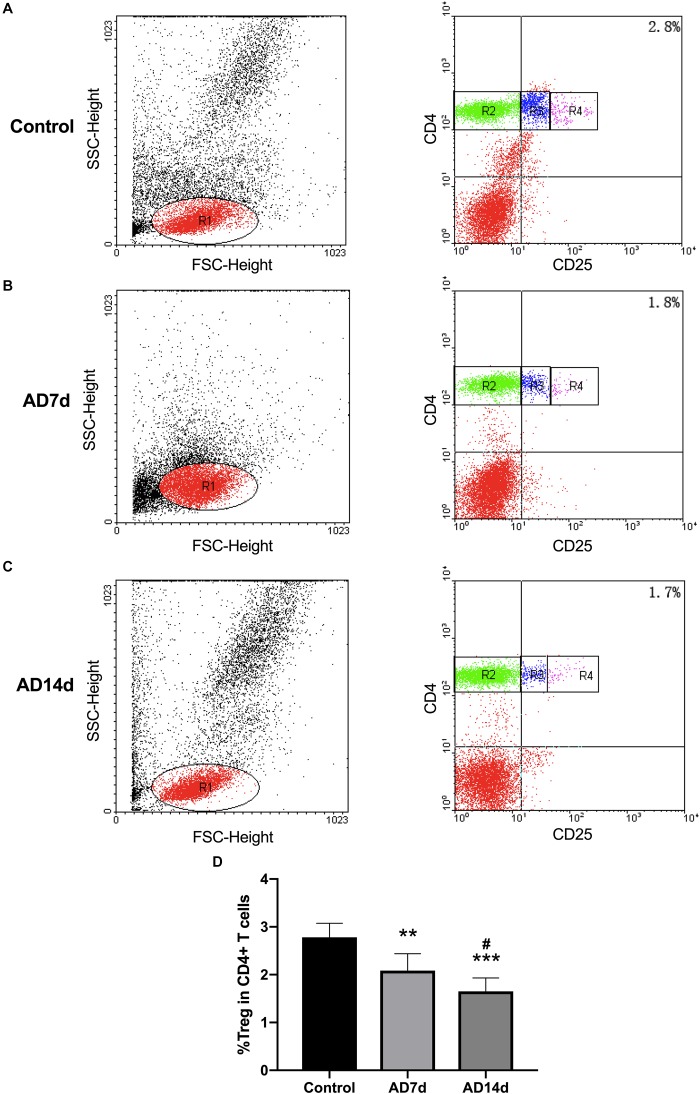
Reduced circulating Tregs populations in AD rats. The population of Tregs, as a subset percent of total CD4+ cells, was estimated with use of flow cytometry. Dot plots shown are representative of rats in the Control **(A)**, AD7d **(B)**, and AD14d **(C)** groups. Tregs were confirmed as CD4^+^CD25^high^ T cells. Numbers in each compartment indicate the proportion of Tregs in CD4^+^ cells. **(D)** Percent of Tregs in total CD4+ T cells in the Control, AD7d and AD14d groups. *n* = 9 per group, mean ± standard deviation, ^∗∗^*P* < 0.01, ^∗∗∗^*P* < 0.001 vs. Control group, #*P* < 0.05 vs. AD7d group.

Populations of Th17 and Tregs subsets, as a percent of total CD4+ T lymphocytes, were detected with use of flow cytometric analysis. While the percent of Th17 within the peripheral blood of AD rats was significantly increased (Figure 6), the percent of Tregs was significantly decreased in AD rats (Figure 7) as compared with controls. There were also significant differences in Th17 and Tregs proportions between rats from the AD7d versus AD14d group (Figure 6D, 7D) These results demonstrate that the balance of Th17/Tregs was disrupted following an intracerebroventricular administration of Aβ_1–42_.

### Correlations Between Netrin-1 and IL-17/IL-10 in AD Rats

**FIGURE 8:**
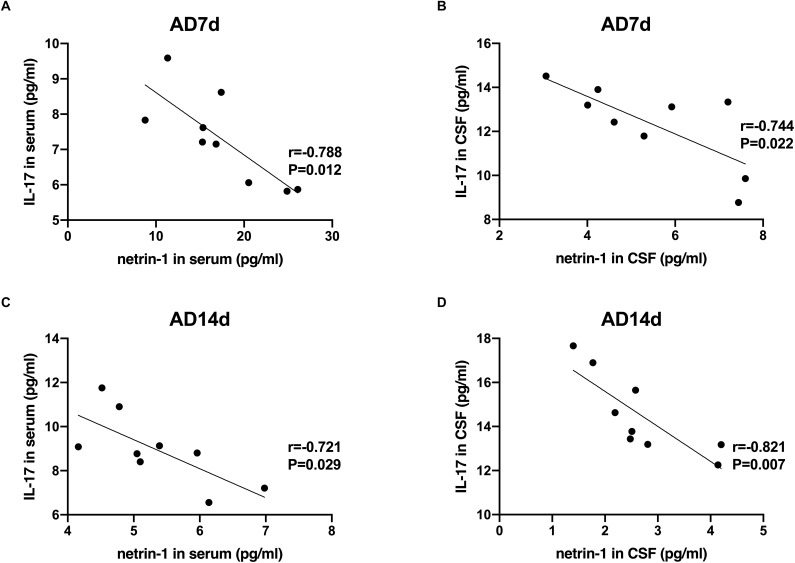
Negative correlations between netrin-1 and IL-17 concentrations in the serum and CSF of AD rats. Negative correlations were obtained between serum netrin-1 and IL-17 concentrations in the AD7d **(A)** and AD14d **(C)** groups. Negative correlations were obtained between CSF netrin-1 and IL-17 concentrations in the AD7d **(B)** and AD14d **(D)** groups.

**FIGURE 9:**
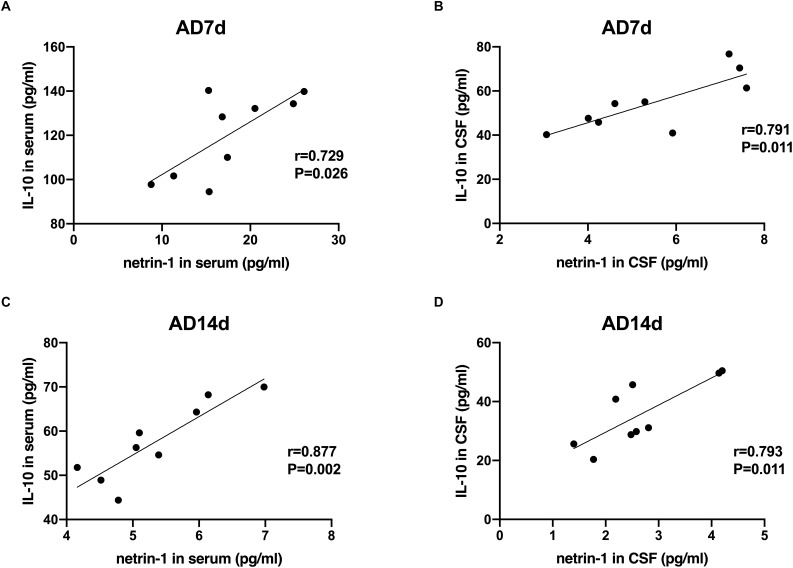
Positive correlations between netrin-1 and IL-10 concentrations in the serum and CSF of AD rats. Positive correlations were obtained between serum netrin-1 and IL-10 concentrations in the AD7d **(A)** and AD14d **(C)** groups. Positive correlations were obtained between CSF netrin-1 and IL-10 concentrations in the AD7d **(B)** and AD14d **(D)** groups.

A statistically significant negative correlation was obtained between netrin-1 and IL-17 concentrations in the serum and CSF of both the AD7d and AD14d rats (Figure 8). A positive correlation was observed between netrin-1 and IL-10 concentrations in the serum and CSF of both the AD7d and AD14d groups (Figure 9).

## Discussion

Neuroinflammation plays a critical role in AD pathogenesis. However, the changes and role of inflammation with regard to the regulation of netrin-1 in the pathogenesis of AD remains unknown. Based on results obtained in other disease models, netrin-1 has been suggested to regulate the differentiation of Th17 (Tadagavadi et al., 2010) and Tregs (Khan et al., 2011). In the present study, we investigated potential changes in netrin-1 and Th17/Tregs balance, as well as the relationship among netrin-1, Th17/Tregs and cognitive parameters within a rat model of AD. We found that netrin-1 was significantly decreased both in the serum and CSF of AD rats, and positively correlated with the percent of time spent within the target quadrant. Netrin-1 was also found to be negatively correlated with IL-17 and positively correlated with IL-10 in these AD rats. Increased IL-17 levels were negatively while decreased IL-10 levels were positively correlated with the percent of time AD rats spent in the target quadrant.

Injection of Aβ_1–42_ peptide into the brain induces pathological changes resulting in cognitive impairment and behavioral deficits indicative of AD (Eisele et al., 2010). While intracerebroventricular (ICV) administration of Aβ_1–42_ oligomers represents an established approach to generate an AD model primarily resulting from inflammation, it is important to note that there are differences in the preparation of Aβ_1–42_ oligomers among different investigators (Mclarnon and Ryu, 2008). For example, although the Aβ_1–42_ oligomer could be detected within the hippocampus as early as 5 min after ICV injection, these levels were significantly increased after 60 min according to the procedures of Kasza et al. (2017). In that study, the Aβ_1–42_ oligomers were prepared by incubating the Aβ_1–42_ peptide in PBS at 37°C, pH 7.4 for 24 or 168 h. In another study, Aβ plaques were detected within both the cortex and hippocampus of Wistar rats after ICV injection of Aβ_1–42_, which was prepared in PBS and incubated at 37°C for 7 days prior to use, a procedure which was similar to that used in our present study (Yaghmaei et al., 2014). However, after chronic administration of Aβ_1–42_ oligomers for 4 weeks in male Long Evans rats using a different preparation process, no Aβ plaques were observed within the hippocampus (Fekete et al., 2018). Accordingly, while Aβ_1–42_ plaques may not always be detectable after icv administration, the inflammatory and behavioral changes of AD remain present. Following Aβ_1–42_ administration we observed that rats showed delayed escape latencies during cued learning trials, and a decreased percent of time within the target quadrant and fewer crossings during the probe trial in the MWM test. These deficits were enhanced in the AD14d versus the AD7d group, suggesting that a progression in the neurodegeneration was present in this model, results which were consistent with that reported by Zhang et al. (2013). We also found that substantial amounts of neuronal loss were present within the hippocampal CA1 region of these AD rats as compared with that of controls. No statistically significant differences in neuronal loss were observed between the AD7d and AD14d groups, which suggests that there may be more progression-related effects responsible for cognitive deficits other than that attributable to direct neuronal loss, such as inflammation.

Netrin-1, a neuronal guidance molecule, has been shown to exert an unexpected immunomodulatory function, demonstrating the ability to inhibit leukocyte migration and alleviate inflammation-mediated tissue injury (Ly et al., 2005; Aherne et al., 2013). Here, we demonstrate a down-regulation of netrin-1 in both the circulation and CNS within AD rats relative to that of controls. This down-regulation was more evident in the AD14d as compared with that in the AD7d group, suggesting that netrin-1 participates in the progression of AD.

We also show that netrin-1 concentrations in the serum from all three groups were positively correlated with the percent of time spent in the target quadrant. More robust correlations were obtained following Aβ_1–42_ administration and as a function of time post-infusion. Within AD rats, CSF netrin-1 concentrations were also positively correlated with the percent of time in the target quadrant, however, no such correlation was observed in Control rats. These results suggest that netrin-1 might be affected by the infusion of Aβ_1–42_. This is in accordance with results reported in a transgenic mouse model of AD, where decreased levels of netrin-1 expression were associated with increased Aβ concentrations (Lourenço et al., 2009). In that study, the authors emphasized the capacity for the netrin-1 to bind with amyloid precusor protein (APP), thus decreasing the concentrations of Aβ_1–40_ and Aβ_1–42_. It has also been reported that netrin-1 reduces Aβ_1–40_-induced Aβ_1–42_ increases as demonstrated *in vitro*, and ICV delivery of netrin-1 to PDAPP^Swe/Ind^ mice decreased Aβ and improved working memory (Spilman et al., 2016). Findings from a recent study have indicated that administration of netrin-1 improved cognitive dysfunction, likely by preventing Aβ_1–42_ induced apoptosis pathways, such as caspase- 3, caspase-7, and NF-κB activation (Zamani et al., 2019). However, little attention has been directed toward examining Th17/Tregs inflammatory mechanisms in AD.

In this study we show that a disrupted Th17/Tregs balance is observed in Aβ_1–42_ induced AD rats. In these AD rats, Th17 is present in higher proportions within CD4+ T cells of the peripheral blood as compared with that in controls, which parallels the cytokine IL-17 levels in both serum and CSF of these AD rats. These increased IL-17 concentrations were consistent with results of a previous study within our laboratory (Zhou et al., 2016) as well as that reported by others (Saresella et al., 2011; Zhang et al., 2013, 2015). Moreover, these serum and CSF IL-17 concentrations were both negatively correlated with the time spent in the target quadrant in AD rats, supporting the hypothesis that Th17 participates in neuroinflammatory processes associated with AD. Interestingly, IL-17 concentrations in the serum and CSF were also negatively correlated with netrin-1 concentrations in these AD rats, suggesting the possibility that an important relationship may exist between these molecules. Results obtained from an *in vitro* study have shown that netrin-1 suppresses IL-17 production from CD4+ T cells via the UNC5B receptor and exogenous netrin-1 also decreased the proportion of Th17 within the CNS of EAE mice (Tadagavadi et al., 2010; Podjaski et al., 2015). Taken together, these findings suggest that reductions in netrin-1 levels may diminish its capacity to inhibit Th17, thus promoting AD pathogenesis.

Conversely, the proportion of Tregs was lower in CD4+ T cells within peripheral blood of AD rats relative to that of controls, with decreased concentrations of IL-10 being observed in both serum and CSF. In addition, serum IL-10 concentrations in Control rats were positively correlated with the percent of time in the target quadrant. Whereas, decreased levels of both serum and CSF IL-10 concentrations in AD rats were positively correlated with the time spent in the target quadrant, correlations which became more robust as a function of time following Aβ_1–42_ treatment. Results from several studies have suggested that Tregs play a protective role in AD progression. For example, systemic transplantation of purified Tregs decreased the deposition of Aβ plaques and ameliorated cognitive deficits in 3xTg-AD mice (Baek et al., 2016); and amplification of Tregs resulted in increased amounts of plaque-associated microglia and a reversal of cognitive deficits in APPPS1 mice. In contrast, depletion of Tregs resulted in earlier onset time and enhancement of the spatial learning deficits (Dansokho et al., 2016). However, there is one report indicating that a transient depletion of Tregs alleviated neuroinflammation and restored cognitive dysfunctions in the 5XFAD AD mouse model (Baruch et al., 2015), leading to the conclusion that breaking immune tolerance by Tregs mitigates AD pathology. Interestingly, a transient depletion of Tregs was followed by subsequent recruitment of Tregs within the CNS and elevated levels of hippocampal IL-10, suggesting that systemic and CNS Tregs may play different roles in inflammation, an eventuality that requires further investigation. Our current research shows that lower proportions of Tregs/CD4+ T cells within the circulation, along with decreased IL-10 levels in both serum and CSF of AD rats, appear to contribute to a deficiency of Tregs immunosuppression in AD.

We found that IL-10 concentrations were positively correlated with netrin-1 concentrations in the serum and CSF of AD rats. To the best of our knowledge no direct evidence exists demonstrating that netrin-1 promotes Tregs differentiation *in vitro*. Nevertheless, a systemic administration of netrin-1 delivered to low-density lipoprotein receptor knockout mice substantially increased Tregs within the aorta and suppressed immune responses, suggesting that netrin-1 produces an up regulation of Tregs (Khan et al., 2011). In EAE mice, the protective effects of netrin-1 may result from different sources. For example, in addition to its capacity to decrease Th17 in the CNS, the protective effect of netrin-1 may also be related to the increased proportion of IL-10-secreting T lymphocytes in secondary lymphoid tissues (Podjaski et al., 2015). As a result, netrin-1 may initially regulate Th17/Tregs balance and then modulate pathological effects of neuroinflammation.

Although a number of limitations exist in this study, the findings provide some novel insights indicating an inflammatory perspective for the mechanisms of netrin-1 in AD. Moreover, these current findings serve as a foundation for future work directed at examining the significance of Th17/Tregs balance and the underlying signaling pathways resulting from netrin-1 administration in animal models of AD.

## Conclusion

In this study we demonstrate that netrin-1 was reduced and correlated with disrupted Th17/Tregs balance in AD rats. Increased IL-17 and deceased IL-10 levels were observed in both serum and CSF and were correlated with disease severity. Taken together, we propose that in AD pathogenesis, decreasing levels of netrin-1 may result in a diminished capacity for immunosuppressive effects on Th17/Tregs. Such effects suggest further investigations into the potential therapeutic use of netrin-1 in the treatment of AD.

## Ethics Statement

All experimental protocols in this study were approved by the ethics committee of Harbin Medical University.

## Author Contributions

LC and LS: study design and manuscript writing. TJ and TW: TUNEL staining and ELISA. LZ and RA: data analysis. YDL and XZ: induction of AD rat model. FD and YZ: Morris water maze. YS and YL: flow cytometric analysis.

## Conflict of Interest Statement

The authors declare that the research was conducted in the absence of any commercial or financial relationships that could be construed as a potential conflict of interest.
